# Herbarium specimens reveal the footprint of climate change on flowering trends across north-central North America

**DOI:** 10.1111/ele.12135

**Published:** 2013-06-21

**Authors:** Kellen M Calinger, Simon Queenborough, Peter S Curtis

**Affiliations:** Department of Evolution, Ecology and Organismal Biology, The Ohio State UniversityColumbus, OH, USA

**Keywords:** Climate change, invasive species, life history, phenological responsiveness, phenology, pollination syndrome

## Abstract

Shifting flowering phenology with rising temperatures is occurring worldwide, but the rarity of co-occurring long-term observational and temperature records has hindered the evaluation of phenological responsiveness in many species and across large spatial scales. We used herbarium specimens combined with historic temperature data to examine the impact of climate change on flowering trends in 141 species collected across 116,000 km^2^ in north-central North America. On average, date of maximum flowering advanced 2.4 days °C^−1^, although species-specific responses varied from − 13.5 to + 7.3 days °C^−1^. Plant functional types exhibited distinct patterns of phenological responsiveness with significant differences between native and introduced species, among flowering seasons, and between wind- and biotically pollinated species. This study is the first to assess large-scale patterns of phenological responsiveness with broad species representation and is an important step towards understanding current and future impacts of climate change on species performance and biodiversity.

## Introduction

Phenology, the timing of key life events, is one of the most sensitive biological indicators of climate change (Peñuelas & Filella 2001; Menzel 2002) and its study has become an important tool for understanding the impacts of warming from local to global scales. Shifts in plant phenophases (e.g. budburst, leaf senescence and flowering) over time, correlated with temperature change, have been documented worldwide and are consistent with climate change predictions (Parmesan & Yohe 2003). This evidence of alterations in plant phenology suggests that increased temperatures may already be impacting species performance and modifying within-community interactions (Menzel *et al*. 2006; Rosenzweig *et al*. 2008).

However, phenological responses to increased temperature are highly variable, even among closely related species (Abu-Asab *et al*. 2001; Fitter & Fitter 2002). For example, in North America, *Solidago rugosa* advanced flowering by ∼ 11 days °C^−1^, while *Solidago graminifolia* showed no temperature response (Miller-Rushing & Primack 2008). In the United Kingdom, *Geranium pyrenaicum* advanced its first flowering date (FFD) 3 days between 1954 and 2000 whereas *Geranium rotundifolium* showed a 6 days delay in FFD over the same time period (Fitter & Fitter 2002). As a result of such species-specific differences in phenological responsiveness to temperature shifts, climate change may non-randomly alter community assemblages as species less well adapted to the new temperature regime decrease in abundance (Willis *et al*. 2008).

The magnitude of a species’ phenological response to temperature may have either positive or negative impacts on growth and reproduction depending on various interacting environmental factors. For instance, phenologically responsive species may benefit from greater early season productivity (Kudo *et al*. 2008) leading to an increase in abundance. Mungia-Rosas *et al*. (2011) suggested that phenotypic selection favours earlier blooming species in temperate regions. Alternatively, earlier flowering can increase risk of frost exposure (Inouye 2008) or may lead to pollinator mismatch, the disconnect between pollinator availability and flowering as a result of differing responses to environmental cues (Kudo *et al*. 2008). Both scenarios may lead to decreased local species abundance. In the northeastern US, Willis *et al*. (2008) found that species with weak flowering responses to temperature have disproportionately decreased in abundance over the past 150 years.

Although species-specific phenological responsiveness is likely to have significant fitness implications with future warming, it is impossible to assess responsiveness for all species in a given area. However, certain life history traits may predispose particular functional groups to be more or less phenologically responsive and we may be able to use these traits to predict how currently unstudied species will respond to warming. Further, if plant functional groups do differ in their phenological responsiveness, leading to increasing variance in fitness, communities may be drastically altered by non-random patterns of species removal. Thus, the ability to make generalised predictions based on functional traits would benefit conservation efforts by allowing identification of species most likely to be negatively impacted (in terms of phenological responsiveness) by temperature increase, as well as identifying whole communities that may be at risk.

Despite the potential impacts of climate change on species performance, few studies have included sufficient species to examine patterns of phenological responsiveness among functional groups because of the lack of long-term flowering and temperature datasets that overlap in time and space. The paucity of direct observational data spanning decades to centuries with broad species representation has led to key information gaps on species’ and functional groups’ responses to climate change, making generalised predictions of community responses to climate change challenging. To date, only three studies have directly evaluated phenological responsiveness to climate change among plant functional groups (Fitter *et al*. 1995; Fitter & Fitter 2002; Miller-Rushing & Primack 2008). In these studies, FFDs of species growing under natural conditions were obtained from observational records that ranged from over 150 years (Miller-Rushing & Primack 2008; with several large gaps in the collection record) to less than 40 years (Fitter *et al*. 1995). While differences in phenological responsiveness among plant functional groups were documented, these studies were conducted over relatively small areas and do not allow evaluation of flowering trends across broad spatial scales (but see Lavoie & Lachance 2006).

An alternative to the traditional historic observational dataset is the analysis of plants preserved in herbarium collections (Primack *et al*. 2004; Lavoie & Lachance 2006; Miller-Rushing *et al*. 2006; Robbirt *et al*. 2011; Panchen *et al*. 2012). For example, by extracting flowering date information on specimens from individual plants collected multiple times at the Arnold Arboretum from 1885 to 2002 and combining that data with long-term local temperature records, Primack *et al*. (2004) found a significant advancement in flowering phenology associated with increasing spring temperatures. To determine the validity of herbarium-based methods for evaluating phenological responsiveness, Robbirt *et al*. (2011) calculated the flowering response of *Ophrys sphegodes* to mean spring temperature (March through May) using both direct observations of peak flowering time and herbarium specimens. The herbarium specimens were collected between 1848 and 1958 while the observational data set spanned 1975 to 2006. Phenological response to temperature calculated using herbarium specimens was statistically indistinguishable from the response calculated from direct field observations (Robbirt *et al*. 2011). These initial herbarium-based methods were an important step towards increasing our ability to assess flowering shifts in areas without historic data sets. However, these methods require intensive historic sampling of a single study site as they do not allow for spatial variation in the temperatures paired with individual herbarium specimens. This intensive sampling is not characteristic of most locations and therefore leaves many regions still inaccessible to study.

Here, we introduce a new herbarium-based method that allows the assessment of flowering phenology in large numbers of species collected across broad geographical regions. We evaluated the phenological responsiveness to temperature of 141 species recorded over a period of 115 years across a 116,000 km^2^ region in north-central North America. We quantified differences in responsiveness among plant functional groups defined by flowering season, growth form, pollination syndrome and native status.

We tested a number of hypotheses: (1) spring flowering species are more responsive to temperature change than those flowering in early- or late-summer because advancing phenology among spring flowering species may enhance pollinator availability and increase access to light prior to canopy closure (Elzinga *et al*. 2007; Kudo *et al*. 2008); (2) herbaceous annuals are more phenologically responsive than herbaceous perennials or woody perennials because earlier flowering may benefit annuals through reduced competition for pollinators and early-season productivity gains during favourable weather which are crucial for enhancing fitness in their single growing season (Mungia-Rosas *et al*. 2011); (3) insect-pollinated species respond more to temperature change than wind-pollinated species because insect-pollinated species may respond to selection promoting earlier flowering to maintain pollinator mutualisms (Fitter & Fitter 2002); and (4) introduced species advance flowering more than native species to take advantage of currently unoccupied phenological niches (Wolkovich & Cleland 2011).

Our results support previously published patterns of responsiveness among different seasons of flowering and growth forms but differ from previous work with regard to native status and pollination syndrome. Our analysis of phenological responsiveness among many species and plant functional groups in a variety of locations is an important step towards understanding the impacts of climate change on species performance and biodiversity.

## Methods

To evaluate flowering phenology, we visually examined specimens of plant species collected in the US state of Ohio (*c*. 116,000 km^2^, between 38^o^ and 42^o^ north latitude and 80^o^ and 85^o^ west longitude) from the Ohio State University herbarium. All specimens with greater than 50% of flower buds in anthesis were considered at peak flowering at the time of collection and were included in our analysis (Primack *et al*. 2004). However, specimens of the same species with identical collection date, location and collector were included as a single datum to avoid non-independence of samples. Because we included only those specimens at peak flowering, some species, such as grasses, were necessarily excluded.

The date of peak flowering (D_i_) and collection location were recorded from the label attached to each specimen. From the collection location, specimens were assigned to one of the ten climate divisions established by the US National Oceanic and Atmospheric Administration (NOAA) for Ohio (See Appendix S1).

We used average monthly temperature data from the US Historical Climatology Network (USHCN, Menne *et al*. 2010) to calculate the average monthly temperature, 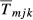, for each climate division in each year;

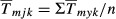
(1)where *m* is month, *j* is climate division, *k* is year, *y* is a recording station within a division, and *n* is the number of recording stations within division *j*. Of the 10 climate divisions in Ohio, all but one had multiple USHCN climate stations. Thus, 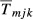 is the average of individual USHCN recording station temperatures within a climate division. Locations of Ohio’s 26 USHCN stations are situated to minimise urban heat island effects and have remained constant since 1895, thus making their data particularly useful for climate change studies.

### Phenological responsiveness

To determine the effect of temperature on flowering date, or a species’ phenological responsiveness (*ρ*_*x*_, day °C^−1^), we regressed D_*i*_ against the average temperature of each specimen’s month of flowering and the 3 months prior (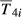), for that specimen’s year and climate division of collection. That is,

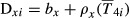
(2)where D_*xi*_ is the flowering date of specimen *i* in species *x*, 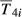 is the average temperature of the month of D_*xi*_ and 3 months prior, and ρ_*x*_ is the slope, or temperature effect size, of this relationship. For example, if a specimen was collected on May 26, 1940, in climate division 10, it would be paired with the average combined temperatures of February, March, April and May, 1940 in that climate division (in this example, 5.21 °C, see Appendix S2). The regression model for each species was built from specimens collected in different years and from a variety of climate divisions, with a minimum of 10 specimens required for each regression. To establish the temperature averaging period, 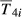, we correlated D_*xi*_ for every species with the average temperature of its month of flowering and the 11 months prior (see Miller-Rushing & Primack 2008 and Appendix S2). We found strong correlations between D_*xi*_ and the average temperature of the month of D_*xi*_ and 3 months prior, and thus used 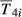 in the regression models. However, as species flowering in April were not strongly correlated with average January temperatures, D_*xi*_ for these species were regressed only against February, March and April monthly average temperatures. Flowering time of species flowering in late-summer were typically not strongly correlated with temperature and were also regressed against 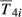. While precipitation is often cited as an important driver of phenology in tropical environments (Rathcke & Lacey 1985), we did not assess the impacts of precipitation on phenological responsiveness as temperature is generally considered the primary variable driving phenology in temperate mesic environments such as our study site. A total of 141 species (*x* = 141) using 5053 specimens (*i* = 5053) were included in our analysis with sample sizes ranging from 10 to 235 specimens per species (see Appendix S3).

### Functional groups

Each species was characterised based on its season of flowering [spring (April to May), early summer (June to July) or late summer (August or later)], pollination syndrome (wind or insect, and facultative or obligate outcrossing), origin (native or non-native to North America) and growth form (woody perennial, herbaceous perennial, herbaceous annual or perennial vine). Information on species’ growth forms was obtained from the USDA Plants Database [http://www.nrs.fs.fed.us/atlas/; Prasad *et al*. (2007)]. Some species exhibit differing growth forms under different environmental conditions and were classified as having multiple growth forms (i.e. herbaceous annual/biennial). Species with a biennial or annual/perennial growth habit were grouped with annuals (as in Fitter & Fitter 2002, see Appendix S3).

Within pollination syndrome, we classified species as insect- or wind-pollinated based on an extensive literature search and the USDA Tree Atlas (http://www.nrs.fs.fed.us/atlas/tree/tree_atlas.html). Insect-pollinated species were further classified as facultative or obligate out-crossers. Obligate out-crossing species are completely self-incompatible while facultative out-crossing species are capable of selfing.

All 141 species were classified according to growth form, flowering time and origin. Thirty-eight species could not be definitively classified with regard to pollination syndrome.

### Statistical analysis

We evaluated differences in the strength of phenological response among functional groups using a linear mixed effects model using the package lme4 of the statistical software R which uses restricted maximum likelihood methods to determine effect sizes (R Development Core Team 2008; Bates *et al*. 2012). Because lme4 does not return *P*-values, we performed a likelihood ratio test using maximum likelihood methods for each term in the model to determine significance (Moore 2010). For each functional group, we included a covariate in our initial model of flowering date as a function of temperature, thereby allowing slope to vary with functional trait (eqn 2). To account for the variation in phenological response among species, the model included the species level intercept and slope of 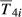 as random effects, thus allowing both the slope and intercept as a function of 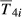 to vary among species.

We examined spatial heterogeneity of phenological responsiveness among climate divisions (Appendix S1). We used a linear mixed effects model with climate division as a covariate added to the initial model of flowering date as a function of temperature with species as a random effect. This model allows the slope to vary between climate divisions and produces mean phenological responsiveness values for each of the 10 climate divisions. We found significant variation in responsiveness only among climate divisions 6 and 7 suggesting little spatial heterogeneity in phenological responsiveness (Appendix S1, Fig. 2).

We further assessed the impacts of spatial variation in our models of functional trait heterogeneity in phenological responsiveness by adding Division, along with species, as random effects to these initial models. This model allows the slope of 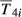 and the intercept to vary both among species and divisions. Our models were not significantly altered by adding Division as a random effect, suggesting that spatial heterogeneity adds no unexplained variation to our functional group analyses (Appendix S1, Figs 3 and 4). Thus, Division was not included in the final models.

Because of their shared evolutionary history, closely related species are likely to be more similar than those species less closely related, leading to non-independence of data and violation of the assumptions of statistical tests in comparative analyses that do not account for this autocorrelation (Revell *et al*. 2008). To test whether significant phylogenetic autocorrelation was present in the phenological responsiveness of species, we first generated a phylogenetic tree of our study species using the software Phylomatic (Stevens 2004; Webb & Donoghue 2005; see Appendix S4). The influence of phylogenetic non-independence on phenological responsiveness was modelled by incorporating the phylogenetic covariance matrix in a generalised least squares model. The phylogenetic covariance structure was multiplied by a phylogenetic signal value (λ), ranging from 0 (no phylogenetic autocorrelation) to 1 (maximum phylogenetic autocorrelation), and the log-likelihood of each run was recorded; from the resulting likelihood surface a maximum likelihood phylogenetic signal value of λ was obtained (Pagel 1999). λ measures the degree to which the variation/covariation of traits across a tree agrees with the Brownian process (Freckleton *et al*. 2002). A value of λ = 0 implies that phenology is distributed among species at random with respect to phylogeny. A value of λ = 1 indicates that phenology is phylogenetically conserved; that is, closely related groups have more similar flowering times than would be expected by chance. Approximate confidence intervals for the maximum likelihood value of λ were calculated via likelihood ratio tests (Freckleton *et al*. 2002) on values derived from the likelihood surface. We used the maximum likelihood method ‘pgls.profile’ function in the R package ‘caper’ to estimate the likelihood profile for λ for species’ phenological responsiveness (Orme *et al*. 2012, see Appendix S5).

## Results

**Figure 1:**
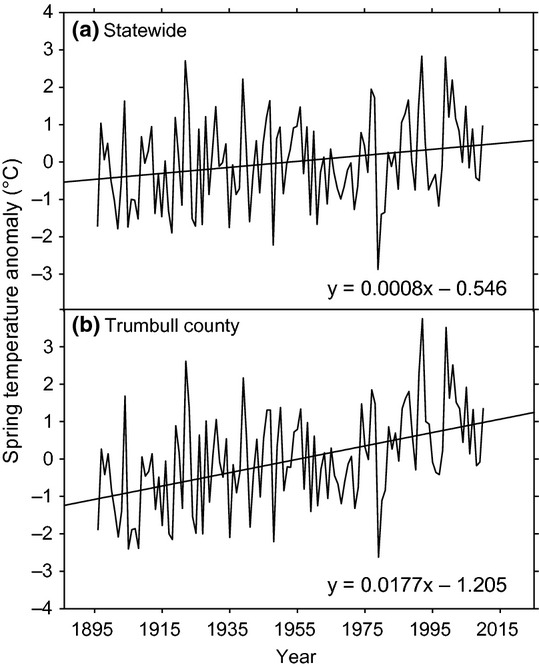
Spring temperature trends across the state of Ohio (a) and in Trumbull County (b). Yearly spring (February to May) temperature anomalies were calculated by subtracting the 115-year (1895–2009) average for these four months from yearly averages. Simple linear regression lines were added to determine shifts in spring temperatures over the 115-year period. Trumbull Co. has experienced a 2.0 °C average spring temperature increase compared with the state-wide 0.9 °C average increase.

Average spring temperatures (February to May) across Ohio have increased by 0.9 °C (*P* ≤ 0.01) since 1895 (Fig. 1a) with some areas experiencing warming of up to 2 °C (Fig. 1b). Seventeen of the 26 counties within Ohio with USHCN weather stations recorded a significant warming trend in the spring. Temperature trends for the late summer to early fall (June to September) were more variable with no significant state-wide changes over the past 115 years. Ten counties reported a significant cooling trend since 1895 (average −1 °C) for late summer to early fall, two showed a significant warming trend (average 0.8 °C), and 14 showed no significant change.

**Figure 2:**
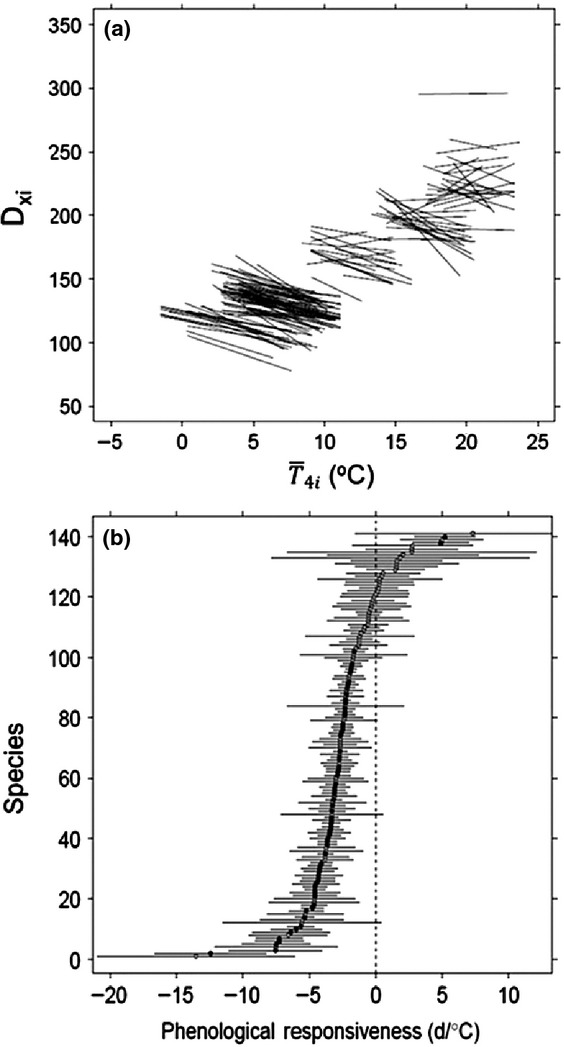
Variation in phenological responsiveness to changing temperature among 141 species of plants in Ohio. (a) For each species, the day of year of maximum flowering (D_*xi*_) was regressed against the average temperature from the average month of flowering and the 3 months prior 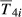. The slope of each line quantifies the phenological responsiveness for each species. (b) Rank order of each species’ phenological responsiveness is represented by a point ± SE bars. Closed points show a significant (*P* ≤ 0.05) phenological response to temperature, while open points designate no significant change. The dashed line indicates 0, or no phenological response. A negative phenological responsiveness indicates earlier flowering with warming while a positive shift represents delayed flowering with warming.

Phenological responsiveness to temperature change showed a high degree of variability among species (Fig. 2). Sixty-six species (46%) showed a significant negative phenological response (i.e. advancement of flowering date with increasing temperature), while only two species (1%) showed a significant positive phenological response (i.e. delay in flowering time with increasing temperature). Among significantly responsive species, *Carduus nutans*, an introduced spring flowering perennial showed the greatest advancement of flowering (−12 days °C^−1^) while *Monotropa uniflora,* a native, early-summer flowering perennial showed the greatest delay in flowering (5 days °C^−1^). On average, flowering advanced 2.4 days °C^−1^ across all species, or 3.7 days °C^−1^ among those species showing a significant negative phenological response. With the increase in spring temperatures state-wide over the past 115 years, flowering may already be 1–3 weeks earlier in particularly responsive species or in areas of locally greater warming.

**Figure 3:**
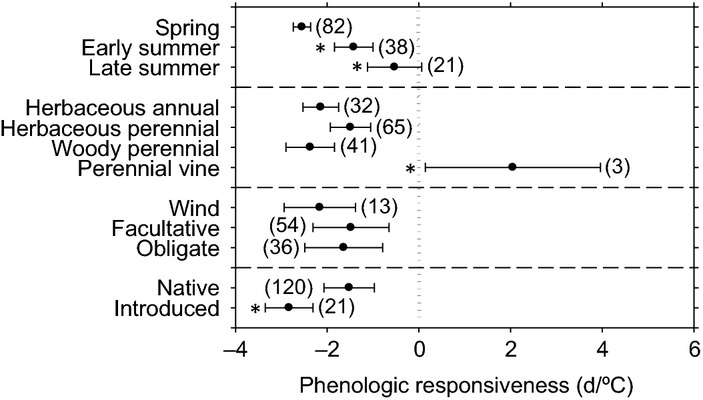
Phenological responsiveness to increasing temperature within different plant functional groups. Mean ± 1 SE with the number of species per group indicated in parentheses. Asterisks indicate a significant difference from the reference group, which is the topmost group in a sub-panel (*P* = 0.05).

Importantly, we found no significant phylogenetic signal in our data (see Appendices S4 and S5). Thus, we can interpret the impacts of functional groups on phenological responsiveness independently from phylogenetic signal. We found significant differences in phenological responsiveness to temperature among functional groups based on seasonality of flowering, growth form and origin (Fig. 3).

There were strong differences in species’ phenological responsiveness based on flowering season. Spring flowering species were significantly more responsive to increasing temperatures (−2.5 days °C^−1^, LR_1_,_9_ = 118.5, *P* < 0.001, Fig. 3) than either early- or late-summer flowering species (−1.4 days °C^−1^, LR_1,9_ = 6.1, *P* = 0.014, and −0.6 day °C^−1^, LR_1,9_ = 10.4, *P* = 0.001; Fig. 3). Given these differences we then evaluated the remaining functional group responses separately for spring, early-summer, and late-summer flowering species. There were few significant differences among functional groups in the early- or late-summer, and thus we focused on between-group differences in spring-flowering species (Appendix S6, Tables 2.1–2.3).

**Figure 4:**
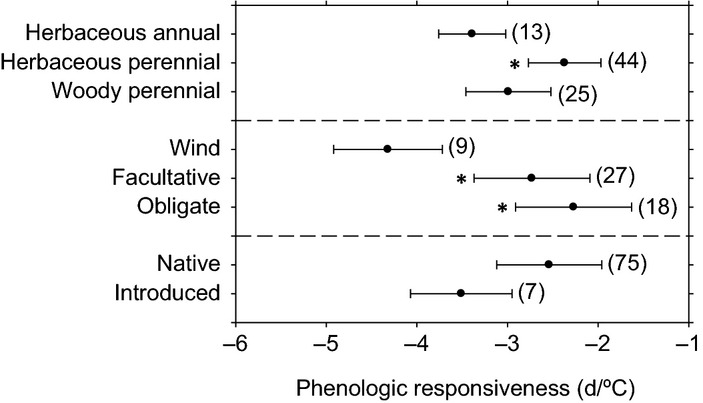
Phenological responsiveness to rising temperature among spring flowering species separated by functional group. Points indicate group mean phenological responsiveness with standard error bars; the number of species included in a group is given in parentheses. Groups that are significantly different from the reference group (the topmost group in a sub-panel) are shown with asterisks (*P* = 0.05).

Growth form also played a role in a species’ phenological responsiveness, with herbaceous annuals and woody perennials showing a similar, strong negative phenological response to increased temperature (herbaceous annuals: −2.2 days °C^−1^, LR_1,11_ = 25.0, *P* < 0.001; woody perennials: −2.4 days °C^−1^, LR_1,11_ = 0.2, *P* = 0.68; Fig. 3). Herbaceous perennials had a weaker phenological response of −1.5 days °C^−1^ compared with woody perennials and annuals, although this difference was not significant (LR_1,11_ = 2.38, *P* = 0.12). In contrast to the other growth form groups, perennial vines delayed flowering with increased temperatures although this response was not significant (2.1 days °C^−1^, LR_1,11_ = 4.8, *P* = 0.028). However, the three perennial vine species in our dataset were all early- or late-summer flowering species, and thus growth form was confounded with seasonality for these species. Further, there was significant variability in phenological responsiveness among growth forms during the spring (Fig. 4). Herbaceous annuals showed the strongest spring flowering response of −3.4 days °C^−1^ (LR_1,9_ = 66.1, *P* < 0.001), and were significantly more responsive than herbaceous perennials, which displayed the weakest response of −2.4 days °C^−1^ (LR_1,9_ = 6.9, *P* = 0.008). Woody perennials shifted flowering −2.9 days °C^−1^ and were not significantly different from annuals or herbaceous perennials (LR_1,9_ = 1.0, *P* = 0.325).

There were no significant differences in phenological responsiveness among different pollination syndromes across seasons (Fig. 3). However, pollination syndrome significantly impacted phenological responsiveness of spring flowering species (Fig. 4). Spring flowering wind-pollinated species had the strongest phenological responsiveness of −4.3 days °C^−1^ (LR_1,9_ = 49.6, *P* < 0.001) compared to the weaker responses of facultative and obligate out-crossing insect-pollinated species (facultative: −2.7 days °C^−1^, LR_1,9_ = 6.1, *P* = 0.014, obligate: −2.3 days °C^−1^, LR_1,9_ = 9.5, *P* = 0.002 respectively).

**Figure 5:**
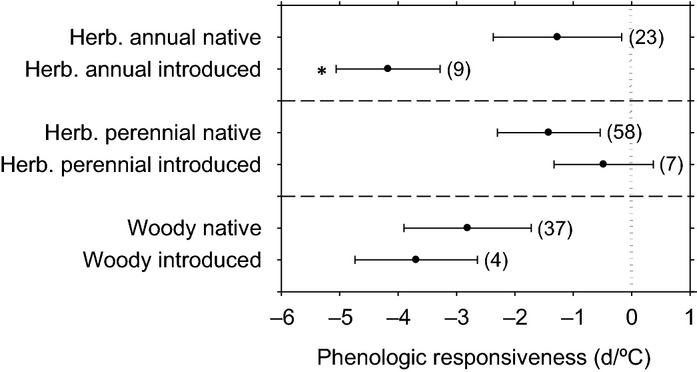
Differing phenological responsiveness to temperature based on origin among differing growth forms. Group mean phenological responsiveness is indicated by points and ± 1 SE bars. Species numbers per group are given in parentheses; significant differences between groups are indicated with asterisks (*P* = 0.05).

Introduced species were almost twice as responsive in advancing flowering time with temperature (−2.8 days °C^−1^, LR_1,7_ = 26.9, *P* < 0.001) compared to native species (−1.5 days °C^−1^, LR_1,7_ = 5.9, *P =* 0.015, Fig. 3). The three most phenologically responsive species in our data set, *Datura stramonium* (*−*13.5 days °C^−1^), *Carduus nutans* (−12.5 days °C^−1^) and *Trifolium pratense* (−7.6 days °C^−1^) all were non-natives. Further, the effect of place of origin was even more pronounced among herbaceous annuals, with introduced species of that group advancing flowering 4.2 days °C^−1^ vs. 1.3 days °C^−1^ in native species (LR_1,7_ = 6.87, *P* = 0.01, Fig. 5). We found no significant difference between native and non-native species among herbaceous or woody perennials (Fig. 5). When comparing only spring flowering species, introduced species were not significantly more responsiveness than native species (introduced: −3.5 days °C^−1^, native: −2.5 days °C^−1^, LR_1,7_ = 2.9, *P* = 0.08; Fig. 4).

## Discussion

This study is the first to assess large-scale patterns of phenological responsiveness with broad species representation. Among the 141 species studied, 46% showed significant advancement of flowering phenology with higher temperatures. Considering the average 0.9 °C temperature increase across Ohio over the past 115 years, this suggests that climate change is already altering our ecosystems by causing some species to flower as much as 3 weeks earlier while others show no change. For example, in Trumbull County, where average spring temperatures have increased by 2 °C, *Phacelia purshii* now flowers 2 weeks earlier while *Cypripedium acaule* advances flowering by only 1 week and *Cardamine diphylla* shows no change.

Along with species-specific differences, we found distinct patterns of phenological responsiveness among functional groups. Our results are a significant addition to the limited number of studies worldwide that have evaluated phenological responsiveness at the functional group level. By expanding our understanding of how functional traits shape group-level responsiveness in a novel region as well as across broad spatial scales, we are now better able to predict species-specific and community scale responses to climate change. Given the direct reproductive consequences of phenology shifts, selection for or against phenologically responsive species with future climate warming may result in an unbalanced loss or gain of species sharing certain functional characteristics.

Overall, spring flowering species showed higher phenological responsiveness to temperature than did early- and late-summer flowering species. Earlier onset of growth and flowering for spring species may allow increased light availability by extending the growth and reproductive periods before canopy closure (Kudo *et al*. 2008). Fitter & Fitter (2002) also found that spring flowering species were most sensitive to temperature increase. Kudo *et al*. (2008) suggested a link between earlier phenology, higher productivity, and reproductive benefits for spring flowering species as a result of greater light availability. However, earlier flowering spring species may not increase productivity if leaf phenology remains stable while flower phenology advances. Advanced flowering for spring flowering species may still improve reproductive success by allowing more seeds to germinate and grow in more favourable conditions before summer droughts. The lower responsiveness of early- and late-summer flowering species to temperature may suggest that these species respond more strongly to other environmental factors such as precipitation. For instance, Jentsch *et al*. (2008) found an average flowering advancement of 4 days in response to 32 days of drought in ten grassland and heath species. In contrast, in an experimental warming study, Sherry *et al*. (2007) found no impacts of water availability on the flowering phenology of twelve grassland species with the exception of *Panicum virgatum*. Overall, precipitation seems to be a stronger factor driving flowering phenology in tropical rather than temperate environments (Rathcke & Lacey 1985).

We found significantly earlier flowering with temperature increase in introduced species than in natives, particularly among herbaceous annuals. Several introduced species showed particularly strong phenological responsiveness including *Carduus nutans* (−12.5 days °C^−1^), *Datura stramonium* (−13.5 days °C^−1^), *Trifolium hybridum* (−6.4 days °C^−1^) and *Trifolium pretense* (−7.6 days °C^−1^). Wolkovich & Cleland (2011) suggest that if high phenological responsiveness among introduced species allows them to adjust more rapidly to warming climate, they may become a greater invasion risk. Phenologically responsive non-natives may shift flowering to occupy previously unfilled niches (the vacant niche hypothesis) or by advancing flowering earlier in the spring before natives flower (priority effects, Wolkovich & Cleland 2011). However, the link between phenological responsiveness and invasiveness remains largely untested. Willis *et al*. (2010) found that non-native species in a 67 km^2^ region of New England advanced flowering significantly more with temperature increase than did native species. In addition, non-native species classified as invasive advanced flowering by roughly 9 days more than non-native non-invasives over the past 100 years (Willis *et al*. 2010). Our similar findings indicate that phenological responsiveness is linked to non-nativeness at small-scales (as in Willis *et al*. 2010) but also across much larger spatial scales. Given previous linkages between phenological responsiveness and invasiveness of non-natives, our research may indicate that introduced species flowering throughout the spring and late summer could utilise flowering advancement to aid invasion. Unlike natives, introduced species maintain high phenological responsiveness throughout the late summer, potentially shifting their flowering before unfavourable autumn weather begins.

We found that spring flowering wind-pollinated species were more phenologically responsive than biotically pollinated species. In contrast to our findings, Fitter & Fitter (2002) found greater advancement of flowering with increased temperature among biotically pollinated species with a weaker response among wind-pollinated species. Strong phenological responsiveness to temperature change among wind-pollinated species likely reflects the high benefit of releasing pollen before trees leaf out in the spring allowing greater pollen dispersal through the leafless canopy (Rathcke & Lacey 1985; Whitehead 1969). Bolmgren *et al*. (2003) found more synchronous flowering times among a variety of wind-pollinated species compared to biotically pollinated species, suggesting a strong response among wind-pollinated species to environmental conditions favouring pollen dispersal (Culley *et al*. 2002; Bolmgren *et al*. 2003). Advancing flowering with higher temperatures may be less advantageous for herbaceous wind-pollinated species than for woody species that flower in the canopy. Herbaceous wind-pollinated species may use over-topping, or growing taller than other members of the understory, as the primary means of enhancing pollen dispersal (Bolmgren *et al*. 2003).

We found that herbaceous annuals had the highest average phenological responsiveness in the spring congruent with the results of Fitter & Fitter (2002) and Miller-Rushing & Primack (2008), who both reported greater flowering shift in annuals compared with perennial species. With their single growing season, phenologically responsive annuals may benefit from a rapid onset of productivity and nutrient uptake, possibly allowing increased nutrient allocation to flowers and seeds. Earlier flowering annuals may also experience greater pollinator availability by avoiding peak flowering time and thus increase their reproductive success (Elzinga *et al*. 2007), although the significant advancement of this group may also increase the risk of pollinator mismatch (Kudo *et al*. 2008). This risk would be particularly significant for those obligate out-crossing species. Of the 26 annual species in this study that could be classified regarding pollination syndrome, 18 had selfing ability and would likely have relatively little impact from pollinator mismatch. Along with potential benefits of flowering advancement, highly responsive annual species may also run a significant risk of frost damage. Extreme weather events including unusually high and low temperatures are becoming more frequent and thus, the risk of late season frosts may increase with climate change (Inouye 2008). Frost may be a potent selective force negatively impacting highly responsive annuals as delicate floral structures are susceptible to frost damage (Inouye 2008).

By combining biological and temperature records, we were able to study phenological responsiveness of many species across a broad spatial scale in an area unrepresented by historic observational data sets. Herbarium collections are common at universities and museums throughout the Unites States and contain hundreds to thousands of species collected over long periods of time. Coupled with temperature data from the USHCN, which has 1218 monitoring stations throughout the contiguous US, these records are an untapped wealth of biological information. By using USHCN temperature data to normalise species’ flowering responses across a large area rather than using environmental variables specific to limited areas, our method allows evaluation of phenological responses across much of the Unites States. Using this method, studies of phenological responsiveness to climate change in previously unstudied areas may provide crucial information needed for conservation efforts.

When evaluating phenological responsiveness, particularly with herbarium specimens, we must consider potential errors associated with the choice of the target phenophase and varying flowering duration among species. First-flowering date is commonly chosen as the target phenophase rather than peak flowering time because observing plants for the duration of flowering is challenging and labour intensive. However, assessing FFD can introduce bias resulting from differing population sizes and sampling efforts (Miller-Rushing *et al*. 2008). Our assessment of peak flowering rather than FFD constrains theses effects on responsiveness calculations (Miller-Rushing *et al*. 2008). Evaluation of peak flowering should also limit uncertainty regarding impacts of flowering duration on responsiveness calculations by sampling at a specific point along a potentially broad distribution of flower opening. Further, Primack *et al*. (2004) evaluated differences in flowering shifts derived using herbarium specimens of species with brief (1 week or less), medium (2 weeks) and long flowering durations (3 weeks or more). The mean flowering shifts for each of these groups were not significantly different, indicating that flowering duration did not impact flowering shift calculations. Through our analysis of peak-flowering, it is unlikely that our results are biased by flowering duration, population size fluctuations, or variation in sampling effort over time.

Evaluating species-specific and functional-group level variability in phenological responsiveness is a key step to understanding how climate change is already altering and will continue to alter ecosystems. Our results suggest that future warming will differentially impact plant functional groups. As less responsive species and groups of species are pushed beyond their optimal temperature ranges, performance may decline, decreasing species abundance. Coupled with factors such as habitat loss, decreasing pollinator diversity, introduced species and altered precipitation patterns, a decline in phenologically non-responsive species could represent a major impact on biodiversity.
