# Supplementary material for: Herbarium specimens reveal the footprint of climate change on flowering trends across north-central North America
Source: Ecol Lett. 2013 Jun 21;16(8):1037–44. doi: 10.1111/ele.12135 (PMC3806244; doi:10.1111/ele.12135)
Supplement: Supplementary file 1 [file ele0016-1037-sd1.docx]

Appendix S1. The 10 NOAA climate divisions and locations of the 26 USHCN stations (A-Z) in Ohio and spatial analysis of phenological responsiveness.

*Climate Divisions in Ohio*

The ten climate divisions generally follow an east-west, north-south pattern and were based on crop reporting districts and drainage basins, and then aligned with county borders (Guttman and Quayle 1996). The pairing of specimens with collection-date and collection–location specific temperatures is necessarily somewhat coarse. The number of USHCN weather stations is limited as USHCN stations are required to have a consistent, non-urban location since 1895. Rather than utilizing an alternative temperature data source with more stations, we chose the USHCN data to ensure that false temperature trends as a result of changing station location or monitoring procedures would not skew our results (Keim et al. 2003). Because only 26 counties have USHCN stations, we adopted a climate division approach to pairing specimens with temperatures. While we recognize that this system introduces noise into calculations of phenologic responsiveness, the high percentage of species showing a significant phenologic responsiveness to temperature suggests the noise likely does not impede detection of a biological signal.

Figure 1. The ten NOAA climate divisions in Ohio and the location of the 26 USHCN weather stations (labeled A-Z).

*Spatial Variation in Phenological Responsiveness*

We examined spatial heterogeneity of phenological responsiveness across species by including climate division as a fixed effect covariate interaction with temperature in the main mixed effects model (equation 2 in the main text), testing whether overall phenological responsiveness varied with division. Most climate divisions (2-5, and 8-10) showed no significant difference in phenological responsiveness from division 1 (Fig. 2). Divisions 6 and 7 were significantly more responsive than division 1, with an extra half a d /°C.

We then used climate division as a random effect in the suite of linear mixed effects models used to evaluate function group variation of phenological responsiveness across all species (equation 2 in the main text). This model allowed the slope (phenological responsiveness) and intercept to vary among species as well as among climate divisions, testing whether there was variability in responsiveness among functional groups between climate divisions. We found no significant differences between the original models and those in which Division had been added as a random effect. The relationships between functional characteristics and phenological responsiveness remained unchanged, suggesting that division of collection explains no additional variation in our models and does not impact functional group patterns of phenological responsiveness (Figures 3 and 4).

While analysis of species-level variation should be a subject of future research, our current data set does not permit species-specific assessment of spatial variation in phenological responsiveness because of sample size limitations. Our data set includes 141 species with a minimum required sample size of 10 specimens per species. Applying this same sample size requirement to the assessment of species-level spatial heterogeneity in phenological responsiveness translates to a minimum of 20 specimens per species with at least 10 specimens in two climate divisions. Only 32 species in our data set meet this requirement and only eight of these species were sampled adequately in at least half of the ten climate divisions. Given our limited sample size, species-level analysis of spatial heterogeneity would be more appropriate after the inclusion of additional species in our data set with thorough sampling across the study area and would be more likely to capture any interesting spatial variation.

*Spatial Variation in Climate*

Our study focuses on flowering response to temperature, so we also investigated monthly temperature anomalies for each climate division relative to the statewide mean for a given month

The temperature of most climate divisions generally did not deviate significantly from the statewide mean (Fig. 5). Divisions 5, 8, and 9 were typically warmer than the average while division 3 was cooler. The warm divisions correspond to central/southwestern Ohio and the cool division is northeastern Ohio.

Given the relative uniformity of phenological responsiveness across the state, we find no support for spatial heterogeneity in responsiveness. Those divisions with greater responsiveness do not correspond with anomalously warm or cool divisions, with the exception of division 5.


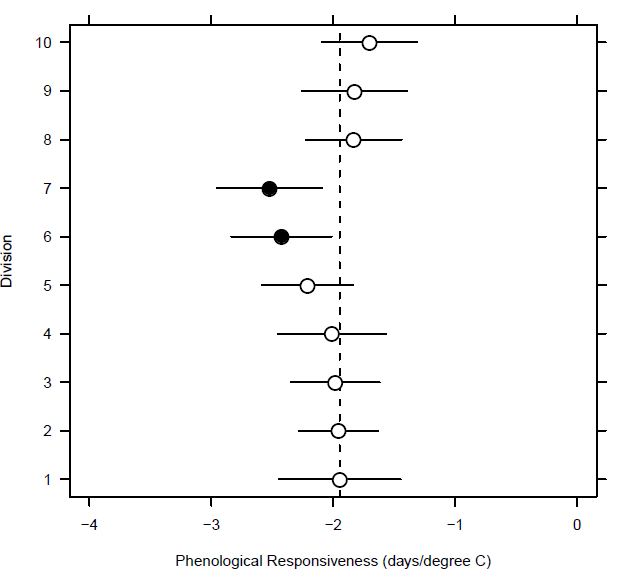
Figure 2. Spatial variability of phenological responsiveness between climate divisions. Mean phenological responsiveness is indicated by each point with standard error bars.


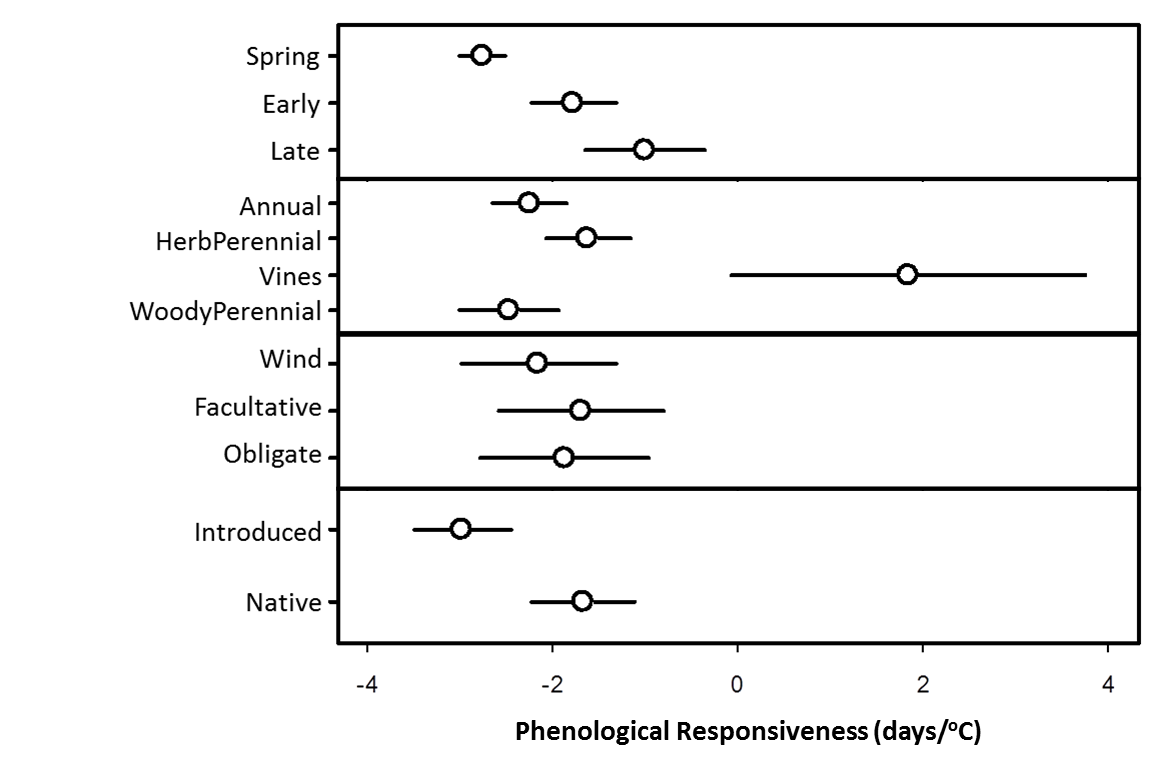


Figure 3. Phenological responsiveness to temperature among functional groups with species and division added as random effects. Points indicate mean phenological responsiveness with +/- standard error bars. All patterns and significant results are maintained from the initial analysis.

Figure 4. Phenological responsiveness to temperature increase among spring flowering species. The patterns and significant effects remain unchanged from the original analysis.

Figure 5. Monthly temperature anomalies from the state mean among the 10 NOAA climate divisions. Average temperature anomalies and standard errors are given for each month for each climate division. Climate divisions are indicated by numbers and correspond to the map in Figure 1 of this Appendix. An anomaly of 0^o^C indicates no difference from the state mean temperature.
