# Supplementary material for: Herbarium specimens reveal the footprint of climate change on flowering trends across north-central North America
Source: Ecol Lett. 2013 Jun 21;16(8):1037–44. doi: 10.1111/ele.12135 (PMC3806244; doi:10.1111/ele.12135)
Supplement: Supplementary file 2 [file ele0016-1037-sd2.docx]

Appendix S2. Calculation of species-specific phenologic responsiveness using *C. acaule* and *C. americana* and correlations between flowering time and average monthly temperature. For each specimen preserved on a herbarium sheet with at least 50% of flowers in anthesis, the maximum date of flowering, D_ix_, the location, and year of collection was recorded directly from the herbarium sheet. For *C. acaule*, the average month of flowering is May (Figure 1). Thus, each specimen was paired with the average temperature of February, March, April, and May in its NOAA climate division and its year of collection ($\bar{T}_{4i}$). For example, a specimen collected on May 26 (day 146) in 1940 from climate division 10 is paired with the average February through May temperature of that year in that division (5.21^o^C). We plotted D_ix_ against $\bar{T}_{4i}$ to produce temperature response curves for each species. Phenologic responsiveness (ρ_x_, d/^o^C) is the slope of the line, $D_{\mathrm{ix}}=\rho_{x}*\left( \bar{T}_{4i} \right)+b$, (Figure 1). *C. acaule* showed a significant phenological responsiveness of -3.4 d/^o^C, whereas the flowering advancement of 0.45 d/^o^C in *C. americana* was not significant (Figure 1).

We correlated average monthly temperatures of the average month of flowering and the eleven months prior with flowering time for all 141 species (Figure 2). Spring flowering species (flowering in April and May) typically showed stronger correlations with temperature than early summer flowering species (June and July). Species that flower during the late summer (August and later) were generally not strongly correlated with any monthly temperatures during or preceding flowering.

Figure 1. Example calculation of species-specific phenological responsiveness using *Cypripedium acaule* and *Campanula americana.*

Figure 2. Correlations between flowering phenology and monthly average temperatures. This plot shows the fraction of species in the spring (flowering in April and May, top panel) and early summer (June and July, bottom panel) climate windows that were significantly correlated at *p*<0.05 with average monthly temperatures in their average month of flowering and the 11 months prior. The average month of flowering is denoted by M with M-1 indicating 1 month before, M-2 indicating 2 months before flowering, and so on.
