# Supplementary material for: Herbarium specimens reveal the footprint of climate change on flowering trends across north-central North America
Source: Ecol Lett. 2013 Jun 21;16(8):1037–44. doi: 10.1111/ele.12135 (PMC3806244; doi:10.1111/ele.12135)
Supplement: Supplementary file 4 [file ele0016-1037-sd4.docx]

Appendix S4. The phylogenetic tree used to perform phylogenetic analysis of phenologic responsiveness.
