# Supplementary material for: Herbarium specimens reveal the footprint of climate change on flowering trends across north-central North America
Source: Ecol Lett. 2013 Jun 21;16(8):1037–44. doi: 10.1111/ele.12135 (PMC3806244; doi:10.1111/ele.12135)
Supplement: Supplementary file 5 [file ele0016-1037-sd5.docx]

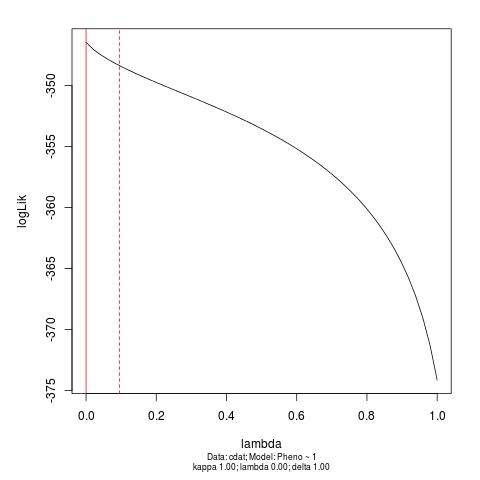


Appendix S5. Analysis of phylogenetic signal in phenological responsiveness. The maximum likelihood estimate of lambda is 0 (solid line, confidence interval = dashed line). Therefore, we find no phylogenetic signal in the data.
