# Supplementary material for: Herbarium specimens reveal the footprint of climate change on flowering trends across north-central North America
Source: Ecol Lett. 2013 Jun 21;16(8):1037–44. doi: 10.1111/ele.12135 (PMC3806244; doi:10.1111/ele.12135)
Supplement: Supplementary file 6 [file ele0016-1037-sd6.docx]

**Appendix S6. Full results tables.**

In this Appendix we provide full tables of the results presented in the main text and Figures 3-5, along with LR and p-values generated for the fixed effects. We use the function p.values.lmer() to calculate p-values, modified slightly to also return the LR values. LR and p-values for each parameter in a model were calculated by comparing the full model and a reduced model without that particular parameter (thus, the difference in degrees of freedom between the models is always 1).

**1 Tests of our hypotheses**

We ran a suite of models to test the four following hypotheses described in the main text:

1. Spring flowering species are more responsive to temperature change than those flowering in early- or late-summer

2. Herbaceous annuals are more phenologically responsive than herbaceous perennials or woody perennials

3. Insect-pollinated species respond more to temperature change than wind-pollinated species

4. Introduced species advance flowering more than native species

Using R, we modeled phenological response as a function of each life history trait in turn, using the basic model DOY ~ $\bar{T}_{4i}$ + Trait + $\bar{T}_{4i}$:Trait + (1|Species).

Each table shows estimates of the intercept (first row) and slope (second row) for the trait level highest (closest to ‘A’) in the alphabet, followed by differences between intercepts for the next levels and the base level, then followed by differences between slopes of the next levels and the base level. For example, Table 1.1 shows an intercept of 142.79 and slope of -2.52 for spring-flowering species (the base level). The intercept for early-flowering species is calculated by 142.79 + 58.89, and the slope by -2.52 + 1.10. ‘LR.value’ and ‘p.value.LRT’ refer to likelihood ratio tests of these differences between intercepts and slopes.

| **1.1 Season** |  |  |  |  |  |
| --- | --- | --- | --- | --- | --- |
|  | Estimate | Std. Error | t value | LR.value | p.value.LRT |
| (Intercept) | 142.79 | 2.11 | 67.63 | 379.62 | 0.00 |
| T_4i_ | -2.52 | 0.19 | -13.55 | 118.52 | 0.00 |
| SeasonEarly | 58.59 | 6.37 | 9.20 | 66.50 | 0.00 |
| SeasonLate | 99.71 | 11.42 | 8.73 | 69.91 | 0.00 |
| T_4i_:SeasonEarly | 1.10 | 0.42 | 2.61 | 6.10 | 0.01 |
| T_4i_:SeasonLate | 1.89 | 0.58 | 3.25 | 10.42 | 0.00 |

| **1.2 Growth form** |  |  |  |  |  |
| --- | --- | --- | --- | --- | --- |
|  | Estimate | Std. Error | t value | LR.value | p.value.LRT |
| (Intercept) | 205.07 | 7.81 | 26.26 | 265.24 | 0.00 |
| T_4i_ | -2.16 | 0.39 | -5.58 | 25.00 | 0.00 |
| FormHerbPerennial | -45.38 | 9.04 | -5.02 | 24.55 | 0.00 |
| FormVinePeren | -41.75 | 35.79 | -1.17 | 1.35 | 0.25 |
| FormWoodyPeren | -41.57 | 9.93 | -4.19 | 17.45 | 0.00 |
| T_4i_:FormHerbPerennial | 0.70 | 0.44 | 1.58 | 2.38 | 0.12 |
| T_4i_:FormVinePeren | 4.22 | 1.90 | 2.22 | 4.80 | 0.03 |
| T_4i_:FormWoodyPeren | -0.22 | 0.53 | -0.42 | 0.20 | 0.66 |
|  |  |  |  |  |  |
| **1.3 Pollination Mode** |  |  |  |  |  |
|  | Estimate | Std. Error | t value | LR.value | p.value.LRT |
| (Intercept) | 154.58 | 13.14 | 11.76 | 85.69 | 0.00 |
| T_4i_ | -2.09 | 0.82 | -2.54 | 6.30 | 0.01 |
| PollinationFacultative | 26.32 | 14.69 | 1.79 | 3.23 | 0.07 |
| PollinationObligate | 20.45 | 15.27 | 1.34 | 1.73 | 0.19 |
| T_4i_:PollinationFacultative | 0.53 | 0.88 | 0.61 | 0.39 | 0.53 |
| T_4i_:PollinationObligate | 0.35 | 0.90 | 0.39 | 0.17 | 0.68 |
|  |  |  |  |  |  |
| **1.4 Native Status** |  |  |  |  |  |
|  | Estimate | Std. Error | t value | LR.value | p.value.LRT |
| (Intercept) | 199.49 | 9.54 | 20.91 | 200.12 | 0.00 |
| T_4i_ | -2.85 | 0.51 | -5.55 | 26.94 | 0.00 |
| NativeNative | -34.87 | 10.16 | -3.43 | 11.30 | 0.00 |
| T_4i_:NativeNative | 1.33 | 0.54 | 2.45 | 5.94 | 0.02 |

**2 Investigating season more fully**

We examined whether species life history traits were correlated with the larger phenological response in spring-, early summer-, and late summer-flowering species.

**2.1 Spring**

| **Life form** |  |  |  |  |  |
| --- | --- | --- | --- | --- | --- |
|  | Estimate | Std. Error | t value | LR.value | p.value.LRT |
| (Intercept) | 152.55 | 4.77 | 31.97 | 225.47 | 0.00 |
| T_4i_ | -3.38 | 0.37 | -9.11 | 66.11 | 0.00 |
| FormHerbPerennial | -10.95 | 5.28 | -2.07 | 4.30 | 0.04 |
| FormWoodyPeren | -9.04 | 5.77 | -1.57 | 2.54 | 0.11 |
| T_4i_:FormHerbPerennial | 1.04 | 0.40 | 2.61 | 6.93 | 0.01 |
| T_4i_:FormWoodyPeren | 0.44 | 0.46 | 0.94 | 0.97 | 0.33 |

| **Pollination mode** |  |  |  |  |  |
| --- | --- | --- | --- | --- | --- |
|  | Estimate | Std. Error | t value | LR.value | p.value.LRT |
| (Intercept) | 139.82 | 6.15 | 22.74 | 136.42 | 0.00 |
| T_4i_ | -4.25 | 0.58 | -7.28 | 49.58 | 0.00 |
| PollinationFacultative | 6.41 | 7.00 | 0.92 | 0.88 | 0.35 |
| PollinationObligate | 1.66 | 7.33 | 0.23 | 0.06 | 0.81 |
| T_4i_:PollinationFacultative | 1.53 | 0.61 | 2.48 | 6.09 | 0.01 |
| T_4i_:PollinationObligate | 1.92 | 0.62 | 3.10 | 9.48 | 0.00 |
|  |  |  |  |  |  |
| **Native Status** |  |  |  |  |  |
|  | Estimate | Std. Error | t value | LR.value | p.value.LRT |
| (Intercept) | 149.97 | 6.47 | 23.17 | 177.95 | 0.00 |
| T_4i_ | -3.50 | 0.56 | -6.23 | 34.72 | 0.00 |
| NativeNative | -7.30 | 6.71 | -1.09 | 1.16 | 0.27 |
| T_4i_:NativeNative | 0.99 | 0.58 | 1.73 | 2.93 | 0.08 |

**2.2 Early Summer**

| **Life form** |  |  |  |  |  |
| --- | --- | --- | --- | --- | --- |
|  | Estimate | Std. Error | t value | LR.value | p.value.LRT |
| (Intercept) | 252.55 | 21.29 | 11.86 | 51.79 | 0.00 |
| T_4i_ | -3.64 | 1.20 | -3.02 | 6.35 | 0.01 |
| FormHerbPerennial | -70.71 | 25.88 | -2.73 | 6.12 | 0.01 |
| FormWoodyPeren | -73.47 | 25.44 | -2.89 | 1.60 | 0.01 |
| T_4i_:FormHerbPerennial | 4.07 | 1.49 | 2.73 | 7.45 | 0.02 |
| T_4i_:FormWoodyPeren | 2.91 | 1.52 | 1.92 | 5.95 | 0.05 |

| **Pollination mode** |  |  |  |  |  |
| --- | --- | --- | --- | --- | --- |
|  | Estimate | Std. Error | t value | LR.value | p.value.LRT |
| (Intercept) | 154.61 | 38.42 | 4.02 | 14.45 | 0.00 |
| T_4i_ | 2.07 | 2.25 | 0.92 | 0.91 | 0.34 |
| PollinationFacultative | 36.68 | 42.81 | 0.86 | 0.66 | 0.42 |
| PollinationObligate | 49.13 | 42.04 | 1.17 | 1.08 | 0.30 |
| T_4i_:PollinationFacultative | -2.27 | 2.51 | -0.90 | 0.74 | 0.39 |
| T_4i_:PollinationObligate | -3.41 | 2.47 | -1.38 | 1.47 | 0.23 |

| **Native status** |  |  |  |  |  |
| --- | --- | --- | --- | --- | --- |
|  | Estimate | Std. Error | t value | LR.value | p.value.LRT |
| (Intercept) | 215.52 | 16.25 | 13.26 | 63.28 | 0.00 |
| T_4i_ | -1.90 | 0.95 | -1.99 | 3.34 | 0.07 |
| NativeNative | -36.66 | 20.50 | -1.79 | 3.23 | 0.07 |
| T_4i_:NativeNative | 1.98 | 1.22 | 1.62 | 2.69 | 0.10 |

**2.3 Late Summer**

| **Life form** |  |  |  |  |  |
| --- | --- | --- | --- | --- | --- |
|  | Estimate | Std. Error | t value | LR.value | p.value.LRT |
| (Intercept) | 255.62 | 20.71 | 12.34 | 46.00 | 0.00 |
| T_4i_ | -1.52 | 1.04 | -1.47 | 2.07 | 0.15 |
| FormHerbPerennial | -26.68 | 33.63 | -0.79 | 0.63 | 0.43 |
| FormWoodyPeren | 37.26 | 76.34 | 0.49 | 0.25 | 0.62 |
| T_4i_:FormHerbPerennial | 1.64 | 1.68 | 0.98 | 0.94 | 0.33 |
| T_4i_:FormWoodyPeren | 1.66 | 4.00 | 0.41 | 0.17 | 0.68 |

| **Pollination mode** |  |  |  |  |  |
| --- | --- | --- | --- | --- | --- |
|  | Estimate | Std. Error | t value | LR.value | p.value.LRT |
| (Intercept) | 222.34 | 64.92 | 3.43 | 9.34 | 0.00 |
| T_4i_ | 1.48 | 2.88 | 0.51 | 0.26 | 0.61 |
| PollinationFacultative | 38.54 | 68.03 | 0.57 | 0.33 | 0.56 |
| PollinationObligate | -46.00 | 80.40 | -0.57 | 0.33 | 0.57 |
| T_4i_:PollinationFacultative | -3.10 | 3.03 | -1.02 | 1.06 | 0.30 |
| T_4i_:PollinationObligate | 1.20 | 3.66 | 0.33 | 0.11 | 0.74 |

| **Native status** |  |  |  |  |  |
| --- | --- | --- | --- | --- | --- |
|  | Estimate | Std. Error | t value | LR.value | p.value.LRT |
| (Intercept) | 273.02 | 81.02 | 3.37 | 11.23 | 0.00 |
| T_4i_ | -2.76 | 3.86 | -0.71 | 0.50 | 0.48 |
| NativeNative | -31.50 | 82.83 | -0.38 | 0.13 | 0.72 |
| T_4i_:NativeNative | 2.24 | 3.95 | 0.57 | 0.30 | 0.59 |

**3. Investigation of Native vs. Introduced Species among Growth Forms**

We investigated differences in phenological responsiveness between natives and introduced species among growth forms. We did not perform a similar analysis between pollination syndromes due to inadequate sample sizes.

| **Herbaceous annuals** |  |  |  |  |  |
| --- | --- | --- | --- | --- | --- |
|  | Estimate | Std. Error | t value | LR.value | p.value.LRT |
| (Intercept) | 226.37 | 22.41 | 10.10 | 47.65 | 0.00 |
| T_4i_ | -4.17 | 0.89 | -4.69 | 14.39 | 0.00 |
| NativeNative | -22.94 | 27.17 | -0.85 | 0.76 | 0.38 |
| T_4i_:NativeNative | 2.90 | 1.10 | 2.64 | 6.87 | 0.01 |

| **Herbaceous perennials** |  |  |  |  |  |
| --- | --- | --- | --- | --- | --- |
|  | Estimate | Std. Error | t value | LR.value | p.value.LRT |
| (Intercept) | 189.39 | 14.08 | 13.45 | 98.22 | 0.00 |
| T_4i_ | -0.48 | 0.85 | -0.56 | 0.32 | 0.58 |
| NativeNative | -36.68 | 14.48 | -2.53 | 6.20 | 0.01 |
| T_4i_:NativeNative | -0.94 | 0.88 | -1.08 | 1.12 | 0.29 |

| **Woody perennials** |  |  |  |  |  |
| --- | --- | --- | --- | --- | --- |
|  | Estimate | Std. Error | t value | LR.value | p.value.LRT |
| (Intercept) | 157.15 | 19.09 | 8.23 | 39.98 | 0.00 |
| T_4i_ | -3.69 | 1.05 | -3.50 | 11.62 | 0.00 |
| NativeNative | 12.30 | 20.09 | 0.61 | 0.39 | 0.53 |
| T_4i_:NativeNative | 0.88 | 1.09 | 0.81 | 0.68 | 0.41 |
